# Development of instant paneer type product from groundnut using microwave dehydration

**DOI:** 10.1002/fsn3.2770

**Published:** 2022-02-11

**Authors:** Atreyee Bal, Om Prakash Chauhan, Arun Kumar Pandey, Anil Dutt Semwal, Avinash Mishra, Mona S. Almujaydil, Hend F. Alharbi, Afnan M. Alnajeebi, Hosam O. Elansary, Eman A. Mahmoud

**Affiliations:** ^1^ Defence Food Research Laboratory Siddarthanagar Mysore India; ^2^ MMICT&BM(HM), Maharishi Markandeshwar (Deemed to be University) Ambala Haryana India; ^3^ 29016 Division of Applied Phycology and Biotechnology CSIR‐Central Salt and Marine Chemicals Research Institute Bhavnagar India; ^4^ Department of Food Science and Human Nutrition, College of Agriculture and Veterinary Medicine Qassim University Buraydah Saudi Arabia; ^5^ Department of Biochemistry, Collage of Science University of Jeddah Jeddah Saudi Arabia; ^6^ Department of Plant Production, College of Food and Agriculture Sciences King Saud University Riyadh Saudi Arabia; ^7^ Department of Food Industries, Faculty of Agriculture Damietta University Damietta Egypt

**Keywords:** dehydration, groundnut, microwave, paneer, vacuum

## Abstract

The present study aimed at the development of a groundnut‐based dehydrated paneer type product which could serve as an instant vegan protein source. In the process of preparing groundnut paneer, a ratio of 1:5 of groundnut to water and 2.0% calcium chloride (CaCl_2_) solution was used for the preparation of groundnut paneer and the product was evaluated for physicochemical, instrumental color, instrumental textural, and sensory characteristics. The developed paneer cubes (1.5 × 1.5 × 1 cm) were dehydrated using a vacuum‐assisted microwave dryer at different microwave powers (200–600 W, 250 mbar vacuum). The minimum bulk density (0.55 g/cc) and maximum rehydration ratio (2.9) were recorded in the sample dehydrated at 600 W. The samples dried at 600 W also showed significantly (*p* < .05) higher L* values, softer texture and high sensory scores for color, aroma, taste, texture, and overall acceptability after rehydration.

## Graphical abstract

Effect of variable microwave drying on rehydration ratio and sensory overall acceptability of groundnut paneer. A groundnut‐based dehydrated paneer type product was developed to serve as a vegan protein source. The power of vacuum‐assisted microwave significantly influenced the rehydration properties of groundnut paneer.

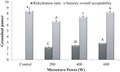

**Practical application**

This study pertains to the development of instant paneer from groundnut for use during culinary preparations. Instant paneer was developed using the vacuum‐assisted microwave dehydration technique to get instant rehydration at the time of use. The dehydrated paneer with high shelf‐life can be used as an alternative to milk paneer.

## 1 INTRODUCTION

The blooming adaptability of plant‐based dairy alternatives considerably increases the interest in the development of milk and milk product substitutes of traditional bovine milk. In recent years, researchers have been focusing on utilizing groundnut (*Arachis hypogaea* L.) milk to convert into low‐cost edible products with a high nutritional value such as yogurt, buttermilk, ripened cheese analogs, and some on fermented products, in particular, the curd or “tofu.” Tofu from soy milk appeared long time ago (Cai et al., 1997; Lim et al., 1990; Shen et al., 1991). Tan et al. (2013) reported on the covariant between the heated time, CaSO_4_ content, and the pressing force with the hardness of soft tofu and that all these factors have a strong impact on the hardness. In addition, recent studies on fermented products’ manufacture have revealed that hexanal, which is one of the compounds responsible for an unwanted nutty flavor in groundnut milk, completely disappeared as a result of fermentation (Diarra et al., 2005). However, cooking at an optimum temperature for a certain time may also reduce the hexanal content of groundnut milk (Omogbai & Jacob, 2013).

Oxidation of lipid fraction is usually involved as a primary cause for a low shelf‐life, off‐flavor, and generation of undesirable aromas during an extended storage of groundnut‐based products (Foffe et al., 2020). Also, groundnuts tend to be contaminated with aflatoxin due to fungal growth (Priya & Chaturvedi, 2020). Chauhan et al. (2015) developed a paneer type product from high‐pressure processed peanut milk and reported an increase in the yield and shelf‐life of the resulted paneer. So, it is important to develop preservation methods for the groundnut‐based paneer type product.

Drying is the most widespread method that considerably reduces water activity, inhibits microbiological growth, decreases physical and chemical reaction rates, and reduces weight and volume of the product, thereby, minimizes the transportation cost and increases the shelf‐life. Among the various types of dryers, vacuum‐assisted microwave drying is putting incredible breadth for the generation of best quality dried products (Ozcan‐Sinir et al., 2019). Under vacuum conditions, microwave as the source of thermal energy rapidly diffuses high‐energy water molecules to the product surface and evaporates into the vacuum chamber. The boiling point of water also lowered down under vacuum. Large vapor pressure gradients created between the interior and the surface of food enhance the water removal rate (Zhou et al., 2018). Thus, it reduces the drying temperature and improves the effectiveness of drying, reduces the chances of oxidative alteration, preserving product color, texture, and flavor pertaining to a good rehydration performance, leading to dehydrated and crunched products with better quality attributes. Cui et al. (2008) investigated microwave vacuum drying as a potential method for obtaining high‐quality dried honey. In this study, the drying curves and the temperature changes of the samples were tested during microwave vacuum drying at different microwave powers and vacuum pressure levels. Vacuum‐assisted microwave drying of potato cubes was done by Chauhan et al. (2015) and they concluded that vacuum‐assisted microwave drying can be employed for the dehydration of potato cubes having good rehydration qualities.

As such, reports on the instant paneer type product from groundnut are not available in the literature, therefore, we undertook the present work to develop an instant paneer type product from groundnut using the vacuum‐assisted microwave dehydration technique and to study the quality characteristics of instant paneer after rehydration.

## 2 MATERIALS AND METHOD

Good quality groundnuts were purchased from a local market in Mysore, India. They were stored at room temperature until further processing.

### 2.1 Preparation of groundnut paneer

For making groundnut paneer, 2 kg of cleaned groundnuts was soaked in water (1:6 ratio) for 6–8 h at room temperature. The outer skin of the groundnuts was then peeled manually and washed thoroughly with water. Deskinned groundnuts were ground in hot water (85°C) using a domestic grinder (Bajaj GX 1) to destroy the lipoxygenase and thus minimize the nutty flavor in the product at different seed‐to‐water ratios (1:3, 1:5, 1:7, and 1:9). The groundnut milk slurry so obtained was filtered through a muslin cloth to separate groundnut milk and heated to 110–115°C with occasional stirring. The temperature was maintained for 15 min followed by cooling. Calcium chloride as coagulant was added in three different concentrations (1.0%, 2.0%, and 3.0%) to groundnut milk at 45°C, followed by continuous stirring at a speed of 30–40 motions per min. A resting time of 20 min was given for complete coagulation and was achieved, as evidenced from the clarity of whey. The obtained whey was then decanted and the coagulated mass was transferred to a perforated tray lined with muslin cloth. The muslin cloth was folded over the top and the coagulated mass was pressed for 30 min. Pressure was applied at the top by placing a weight of 3 kg initially and 1.5 kg for the next 20 min to strain the remaining whey from the resulted product. The obtained groundnut paneer was then stored in a sterilized high‐density polyethylene (HDPE) pouches at 4°C until further processing.

### 2.2 Vacuum‐assisted microwave dehydration

Paneer samples were prepared before each trial run. The paneer was cut into cubes of 1.5 × 1.5 × 1 cm size. Samples were evenly spread and placed as a single layer on the base of a sample holder. A sample of 200 g was used for each experimental run and microwave dehydration of the samples was performed at 200, 400, and 600 W microwave power and at 250 mbar vacuum level. Moisture loss was recorded by taking out the samples and weighing on a digital balance periodically of 0.001 g accuracy until a moisture content of about 5%–6% (wet basis) was reached. Each experiment was replicated three times and the mean value and standard error was calculated in moisture content at each experiment. Later, the dehydrated paneer cubes were stored at room temperature (25°C ± 2°C) in HDPE pouches for further analysis.

### 2.3 Chemical analysis

Moisture, protein, fat, and ash contents were determined using AOAC (2000) methods and the results are reported on % dry basis (*db*). Carbohydrate was calculated by subtracting the sum of moisture, protein, fat, and ash from 100 (Merrill & Watt, 1973). The gross energy value (kcal/100 g) was estimated using the factors for protein (4 kcal/g), fat (9 kcal/g), and carbohydrate (4 kcal/g).

### 2.4 Color measurement

The sample color was measured using D‐65 illuminant and 10° observer using a color meter (MiniScan XE Plus, Model No. 45/0‐S, Hunter Associates Laboratory, Inc.). The CIE (Commission Internationale de 1’Eclairage) color values were expressed as L* (lightness/darkness), a* (redness/greenness), and b* (yellowness/blueness). Standard white and black tiles were used as a reference. Triplicate readings were carried out for each sample and average of the same was reported.

### 2.5 Rehydration

Rehydration characteristics for the dehydrated groundnut paneer were evaluated in triplicate, by immersing 5 g of sample in boiling water for 3 min as per the method given by Ranganna (1999). The samples were removed from water and surface moisture was removed by gently wiping it off with a tissue paper and weighed. Dehydrated groundnut paneer was evaluated for rehydration characteristics from the weight of sample before and after the rehydration.

Rehydration ratio = W_r_ / W_d_.

where,

W_r_ = Weight of the rehydrated sample (g).

W_d_ = Weight of the dried sample (g).

### 2.6 Bulk density

Bulk density was determined as per the equation given below, which is the weight of the dehydrated sample by its respective volume and is reported as g/cc by using the method given by Ranganna (1999).

Bulk density = W_1_/W_2_.

where,

W_1_ = Weight of the dehydrated sample.

W_2_ = Volume of the dehydrated sample (measured using graduated cylinder).

### 2.7 Texture profile analysis (TPA)

Texture profile analysis was carried out with a texture analyzer (TAHDi, Stable Micro Systems Ltd.) using a 25‐kg load cell and the data were recorded with a computer supported with software (Texture Expert, Version 1.22, Stable Micro Systems Ltd.). The samples were compressed to 75% of their original height by two consecutive compressions using a 75‐mm compression plate probe. The crosshead speed was maintained at 1.00 mm/s. The waiting time between the two cycles of the TPA tests was 5 s. The texture analyzer was calibrated for force and height every time before starting the TPA tests. The data were recorded in triplicate and mean values were considered for computing the final results.

### 2.8 Sensory acceptability

The groundnut paneer samples were subjected to sensory acceptability in fresh condition as well as after the rehydration of dehydrated ones. The sensory panel consisted of 30 semi‐trained panelists for judging the overall sensory acceptability based on the visual appearance, color, texture, and taste of the samples on a 9‐point hedonic scale; 9 indicating highly acceptable and 1 as least acceptable (Lawless & Heymann, 2010).

### 2.9 Statistical analysis

All data were reported as mean ± standard deviation of three replicates. The data were analyzed statistically using the analysis of variance (ANOVA) technique to determine significant differences among various treatments at *p* < .05 significance level using Statistica 7 software (StatSoft).

## 3 RESULT AND DISCUSSIONS

### 3.1 Optimization of process for the preparation of groundnut paneer

Different groundnut:water ratios were tested (1:3, 1:5, 1:7, and 1:9) to optimize the milk extraction. Significantly, a decrease in the solid content was observed with the increase in water content in groundnut milk (Table 1). During the extraction of groundnut milk, the amount of water added has a major role, as it directly affects the solid content of milk and quality of paneer, especially the texture of coagulated proteins. There was no significant change in pH and titratable acidity of milk with the increase in amount of water. For the preparation of paneer, the groundnut milk of 5% solid content was prepared using the 1:5 groundnut:water ratio. According to Cai et al. (1997), lower solid content causes higher moisture content in the coagulated proteins resulting in a soft texture. Based on moisture content, protein content, fat content, and final yield, optimization of the calcium chloride concentration as coagulant was carried out. Table 1 shows that a 3% of calcium chloride concentration had a significantly (*p* < .05) higher moisture content than the other concentrations (Table 2). The 3% concentration of CaCl_2_ delivered the maximum yield as compared to other concentrations (Table 2). It might be due to the higher moisture holding capacity of the samples as the yield and moisture contents are highly correlated (Cai et al., 1997). Calcium chloride with 2% concentration shows groundnut paneer yield in the range of 130–138 g/100 g, which is to some extent nearer to the yield of 3% concentration. Therefore, for the preparation of groundnut paneer form 2% CaCl_2_ concentration was selected for further studies. Analyzing the cumulative effect, it was observed that fat content increased significantly (*p* < .05) with the increase in coagulant concentration and amount of moisture present in the paneer (Table 2). However, contrary to our findings, Cai and Chang (1998) also reported an increase in the concentration of coagulant lowers the fat content in tofu. Considering the health benefits, a lower fat content may be preferable; therefore, a higher quantity of the coagulant is required to achieve the lower fat value (Jayasena et al., 2014). An increase in coagulant concentration also resulted in an increase in protein content, which is dependent on the moisture content of the paneer (Table 2).

**TABLE 1:** Effect of water content on the extraction of groundnut milk

Groundnut:water ratio	Milk (ml)	Total soluble solid (°B)	pH	Titratable acidity (%)
1:3	142^d^	7 ± 0.20^a^	6.8 ± 0.04^a^	0.58 ± 0.04^a^
1:5	246^c^	5 ± 0.18^b^	6.8 ± 0.02^a^	0.58 ± 0.01^a^
1:7	345^b^	4 ± 0.36^c^	6.8 ± 0.05^a^	0.58 ± 0.03^a^
1:9	448^a^	2 ± 0.16^d^	6.8 ± 0.03^a^	0.58 ± 0.06^a^

Note

Values with different superscripts in the same column differ significantly (*p* < .05)

**TABLE 2:** Effect of calcium chloride concentration on the preparation of groundnut paneer

Salt concentration (%)	Moisture content (% *db*)	Yield (%)	Protein content (% *db*)	Fat content (% *db*)
1	84.81 ± 0.22^a^	121 ± 7.0^a^	29.42 ± 0.60^a^	38.75 ± 0.08^a^
2	102.92 ± 0.34^b^	134 ± 4.0^b^	34.60 ± 0.52^b^	42.80 ± 0.05^b^
3	136.57 ± 0.62^c^	137 ± 3.0^b^	35.07 ± 0.24^b^	45.23 ± 0.09^c^

Note

Values with different superscripts in the same column differ significantly (*p* < .05)

### 3.2 Physicochemical properties of optimized fresh groundnut paneer

The moisture, protein, fat, ash, fiber, and carbohydrate contents were found to be 103.71%, 34.91%, 42.98%, 3.07%, 0.35%, and 17.69% in optimized ground paneer, which were 6.40%, 38.63%, 46.93%, 3.41%, 3.62%, and 7.41% in raw groundnut, respectively. The total energy in groundnut paneer was found to be 597.22 kcal/100g, which was 606.53 kcal/100 g in raw groundnut. The color values of fresh groundnut paneer in terms of L*, a*, and b* values were recorded as 82.62, 1.52, and 5.58, respectively. Groundnut paneer yield was 1.34 kg from 1 kg of groundnut as per the optimized procedure.

### 3.3 Dehydration characteristics

**FIGURE 1:**
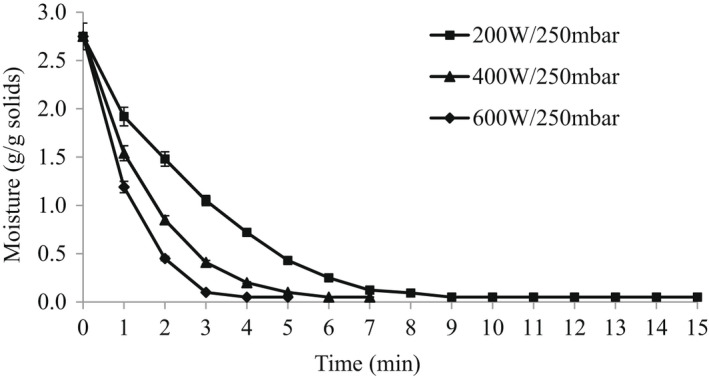
Dehydration curve for groundnut paneer under different drying conditions

Vacuum condition of 250 mbar was used with different microwave power (200 W, 400W, and 600W) conditions to dehydrate groundnut paneer samples. The dehydration curve (Figure 1) for groundnut paneer shows that the drying rate increased exponentially with increase in the microwave power level from 200 to 600 W. Earlier studies also reported that almost all the drying curves of biological materials take place in the falling rate period (Wang et al., 2007). Combination drying techniques have proven to reduce drying time while improving product quality and minimizing energy requirements (Orsat et al., 2006). In vacuum‐assisted microwave drying, electromagnetic energy is directly converted into kinetic energy of the water molecules, thus, generating heat within the product, and energy transport is not affected by conductivity barriers, especially in high‐viscosity materials (Cui et al., 2006). Cui et al. (2006) also demonstrated that microwave power and vacuum pressure affect the drying rate and for constant vacuum pressure, the drying rate was found to be of the first order of microwave power output.

Bulk density was found to be minimum (0.55 g/cc) in the samples dehydrated at 600 W microwave power, whereas, maximum (0.66 g/cc) bulk density was recorded in the sample dehydrated at 200 W microwave power, and at 400W microwave power the bulk density was 0.59 g/cc (Table 3). Bulk density decreases with increase in the level of vacuum acquiring puffed nature of the products (Chauhan, Kumar, et al., 2015). The rehydration ratio is an important parameter as far as the quality of dried products is concerned. This complex procedure indicates the chemical and physical changes caused by drying treatments (Feng & Tang, 1998; Lewicki, 1998). The rehydration ratio was significantly (*p* < .05) higher in the samples dried at higher microwave power conditions (Table 3). The vapor pressure differential between the center and surface of the product might be higher during vacuum microwave dehydration, resulting in a puffy texture of the dehydrated samples (Lin et al., 1998).

**TABLE 3:** Drying characteristics of dehydrated groundnut paneer

Microwave power (W)	Bulk density (g/cc)	Rehydration ratio	Drying time
200	0.66 ± 0.03^a^	2.2 ± 0.02^c^	15 min^a^
400	0.59 ± 0.02^b^	2.5 ± 0.02^b^	5 min^b^
600	0.55 ± 0.02^c^	2.9 ± 0.01^a^	3 min^c^

Note

Values with different superscripts in the same column differ significantly (*p* < .05)

### 3.4 Instrumental color

Color changes are often used as a measurement of quality and freshness for food products. Color changes are very significant when food material undergoes different thermal treatments. Groundnut paneer dehydrated at low microwave power showed significantly (*p* < .05) darker color compared to samples dried at high and intermediate microwave powers (Table 4). These changes may be due to the shorter drying time in the case of higher microwave power levels. Sumnu et al. (2005) reported a similar observation, where, L* values increased with increase in the microwave power level due to the shorter drying time at higher power levels. Chauhan, Kumar, et al. (2015) also observed a similar result in the case of potato cubes. The a* and b* values were also found to be significantly (*p* < .05) higher in the samples dehydrated at high microwave power levels. The rehydrated samples followed a similar pattern as in the case of their dehydrated counterparts. The samples dried at higher power levels showing higher values for L*, a*, and b* values.

**TABLE 4:** Commission Internationale de 1’Eclairage (CIE) color values of groundnut paneer

Sample	Microwave power (W)	L*	a*	b*
Fresh	‐	82.62 ± 1.20^a^	2.52 ± 1.06^a^	5.58 ± 1.12^a^
Dehydrated	200	40.63 ± 1.36^e^	4.93 ± 1.77^b^	23.45 ± 1.03^b^
	400	48.27 ± 1.10^d^	12.04 ± 0.75^f^	31.99 ± 1.50^d^
	600	56.47 ± 1.07^c^	11.55 ± 1.19^e^	48.54 ± 1.21^e^
Rehydrated	200	48.20 ± 1.50^d^	9.03 ± 1.58^d^	30.12 ± 1.63^d^
	400	56.57 ± 1.60^c^	10.34 ± 1.52^d^	29.59 ± 1.21^c^
	600	64.37 ± 1.47^b^	8.34 ± 1.48^c^	23.44 ± 0.80^b^

Note

Values with different superscripts in the same column differ significantly (*p* < .05)

### 3.5 Instrumental textural characteristics

Table 5 shows the textural profile analysis hardness, fracturability, springiness, cohesiveness and gumminess of rehydrated paneer samples. Paneer samples dehydrated under low microwave power (200 W) conditions showed the maximum hardness, whereas, minimum hardness was observed in the samples dehydrated at 600 W. In the case of microwave dehydration, faster drying happened leading to puffed and crisp products (Chauhan, Kumar, et al., 2015). Porosity in texture is facilitated by the insertion of vacuum during microwave dehydration. Texture profile analysis (TPA) of rehydrated groundnut paneer showed significantly (*p* < .05) lower hardness (294.32 g), springiness (1.78 s), cohesiveness (0.63), gumminess (185.42 g), and chewiness (330.04 g.s) when dehydrated at 600W microwave power, which is comparable with the textural characteristics of fresh groundnut paneer.

**TABLE 5:** Texture profile of groundnut paneer

Sample	Microwave power (W)	Hardness (g)	Fracturability (g)	Springiness (s)	Cohesiveness	Gumminess (g)	Chewiness (g.s)
Fresh	‐	224.47 ± 1.21^a^	‐	2.31 ± 1.10^c^	0.62 ± 0.89^a^	255.17 ± 1.03^c^	899.44 ± 0.92^d^
Dehydrated	200	598.54 ± 1.05^e^	34.95 ± 1.11^c^	‐	‐	‐	‐
	400	627.96 ± 1.07	30.43 ± 1.06^b^	‐	‐	‐	‐
	600	632.09 ± 1.01^f^	28.66 ± 1.16^a^	‐	‐	‐	‐
Rehydrated	200	428.47 ± 1.13^d^	‐	2.07 ± 1.17^b^	0.66 ± 1.23^a^	282.79 ± 0.98^d^	585.37 ± 0.85^c^
	400	367.75 ± 1.07^c^	‐	1.91 ± 1.05^a^	0.64 ± 0.87^a^	235.36 ± 1.07^b^	449.53 ± 1.01^b^
	600	294.32 ± 0.96^b^	‐	1.78 ± 0.97^a^	0.63 ± 1.01^a^	185.42 ± 1.03^a^	330.04 ± 0.90^a^

Note

Values with different superscripts in the same column differ significantly (*p* < .05)

### 3.6 Sensory characteristics

Sensory evaluation of fresh and rehydrated groundnut paneer sample was done in terms of color, taste, appearance, and overall acceptability. Sensory acceptability of texture, which is perceived by touching and/or mouth feel, is an important aspect of consumer acceptability (Obatolu, 2008). It can be concluded from the data shown in Table 6 that samples dried at 600W had the highest sensory acceptability and reached nearer to the fresh samples' values after rehydration. It can then be confirmed that the rehydrated groundnut paneer dried by a vacuum‐assisted microwave dryer was highly accepted by the sensory panel and rated as liked very much. Microwave dehydration of groundnut paneer under vacuum conditions showed faster absorption of water when put for rehydration due to porous nature. Chauhan, Kumar, et al. (2015) reported better sensory characteristics in terms of visual appearance, color, texture, and overall acceptability in microwave dehydrated potato cubes with and without vacuum condition that was attributed to faster drying under microwave dehydration condition. According to a report by Fathima et al. (2001), microwave drying also had an effect on the shelf‐life and sensory attributes of coriander, mint, fenugreek, and shepu. Yousif et al. (1999) reported that microwave‐vacuum drying of basil resulted in higher retention of volatiles, better color, and higher rehydration rates in comparison to the conventional hot air method.

**TABLE 6:** Sensory characteristics of groundnut paneer

Sample	Microwave power (W)	Color	Aroma	Taste	Texture	Overall acceptability
Fresh	‐	8.6 ± 0.1^a^	7.6 ± 0.1^a^	7.9 ± 0.2^a^	8.4 ± 0.1^a^	8.5 ± 0.5^a^
Rehydrated	200	6.1 ± 1.1^d^	6.8 ± 0.2^d^	6.5 ± 0.6^c^	6.3 ± 0.2^d^	6.7 ± 0.1^d^
	400	7.6 ± 1.0^c^	7.5 ± 0.2^c^	6.9 ± 0.8^b^	7.0 ± 0.3^c^	7.5 ± 0.1^c^
	600	8.4 ± 1.3^b^	7.5 ± 0.5^b^	7.8 ± 0.9^a^	8.2 ± 0.4^b^	8.3 ± 0.1^b^

Note

Values with different superscripts in the same column differ significantly (*p* < .05)

## 4 CONCLUSION

The results pertaining to the present experiment showed that groundnut paneer prepared from groundnuts can be an alternative to the dairy products. The market value of groundnut paneer depends on the final yield and quality of fresh produce. The groundnut and water ratio of 1:5 was found optimum for the extraction of milk and 2% calcium chloride was found optimum to achieve the desired quality and higher yield of groundnut paneer. Vacuum‐assisted microwave dehydration at 600 W and 250 mbar vacuum could be used for the dehydration of groundnut paneer for the development of an instant paneer type product with excellent rehydration characteristics.

## CONFLICT OF INTEREST

The authors declare that there is no conflict of interest regarding work reported in this paper.

## AUTHOR CONTRIBUTION

**Atreyee Bal:** Investigation (lead); Methodology (equal); Writing – original draft (equal). **O P CHAUHAN:** Resources (equal); Supervision (lead); Writing – review and editing (equal). **A. K. Pandey:** Formal analysis (equal); Methodology (supporting); Writing – review and editing (equal). **Anil Semwal:** Resources (lead); Supervision (supporting). **A Mishra:** Writing – review and editing (supporting). **Mona S. Almujaydil:** Writing – review and editing (supporting). **Hend F. Alharbi:** Writing – review and editing (supporting). **Afnan M. Alnajeebi:** Writing – review and editing (supporting). **Hosam O. Elansary:** Writing‐ reviewing (supporting). **Eman A. Mahmoud:** Funding acquisition (lead); Design Methodology (supporting); Writing – review and editing (supporting).

## Data Availability

The data that support the findings of this study are available from the corresponding author upon reasonable request.

## References

[fsn32770-bib-0001] AOAC (2000). Official methods of analysis of AOAC (17th ed.). Association of Analytical Communities.

[fsn32770-bib-0002] Cai, T. , & Chang, K. (1998). Characteristics of production‐scale tofu as affected by soymilk coagulation method: Propeller blade size, mixing time and coagulant concentration. Food Research International, 31, 289–295. 10.1016/S0963-9969(98)00091-X

[fsn32770-bib-0003] Cai, T. , Chang, K. , Shih, M. , Hou, H. , & Ji, M. (1997). Comparison of bench and production scale methods for making soymilk and tofu from 13 soybean varieties. Food Research International, 30, 659–668.

[fsn32770-bib-0004] Chauhan, O. P. , Bhawya, D. , Kumar, M. , Roopa, N. , & Raju, P. S. (2015). Effect of variable microwave power and vacuum levels during dehydration on the quality attributes of potato cubes. American Journal of Advanced Food Science and Technology, 3, 53–66. doi:10.7726/aiafst.2015.1005

[fsn32770-bib-0005] Chauhan, O. P. , Kumar, S. , Nagraj, R. , Narasimhamurthy, R. , & Raju, P. S. (2015). Effect of high pressure processing on yield, quality and storage stability of peanut paneer. International Journal of Food Science & Technology, 50, 1515–1521.

[fsn32770-bib-0006] Cui, Z. , Sun, L. , Chen, W. , & Sun, D. (2008). Preparation of dry honey by microwave–vacuum drying. Journal of Food Engineering, 84, 582–590.

[fsn32770-bib-0007] Cui, Z. , Xu, S. , Sun, D. , & Chen, W. (2006). Dehydration of concentrated *Ganoderma* *lucidum* extraction by combined microwave‐vacuum and conventional vacuum drying. Drying Technology, 24, 595–599.

[fsn32770-bib-0008] Diarra, K. , Nong, Z. G. , & Jie, C. (2005). Peanut milk and peanut milk based products production: A review. Critical Reviews in Food Science and Nutrition, 45, 405–423. 16130416 10.1080/10408390590967685

[fsn32770-bib-0009] Fathima, A. , Begum, K. , & Rajalaksmi, D. (2001). Microwave drying of selected greens and their sensory characteristics. Plant Foods for Human Nutrition, 56, 303–311. 11678436 10.1023/a:1011858604571

[fsn32770-bib-0010] Feng, H. , & Tang, J. (1998). Microwave finish drying of diced apples in a spouted bed. Journal of Food Science, 63, 679–683.

[fsn32770-bib-0011] Foffe, H. A. K. , Houketchang, S. C. N. , Djikeng, F. T. , Teboukeu, G. B. , Tsopmo, A. , & Womeni, H. M. (2020). Effect of *Syzigium aromaticum* and *Allium sativum* spice extract powders on the lipid quality of groundnuts (*Arachis hypogaea*) pudding during steam cooking. Heliyon, 6, e05166. 33088949 10.1016/j.heliyon.2020.e05166 PMC7566938

[fsn32770-bib-0012] Jayasena, V. , Tah, W. , & Nasar‐Abbas, S. (2014). Effect of coagulant type and concentration on the yield and quality of soy‐lupin tofu. Quality Assurance and Safety of Crops & Foods, 6, 159–166.

[fsn32770-bib-0013] Lawless, H. T. , & Heymann, H. (2010). Sensory Evaluation of Food. Food Science, 2nd ed. Springer‐Verlag.

[fsn32770-bib-0014] Lewicki, P. P. (1998). Some remarks on rehydration of dried foods. Journal of Food Engineering, 36, 81–87.

[fsn32770-bib-0015] Lim, B. T. , DeMAN, J. M. , DeMAN, L. , & Buzzell, R. I. (1990). Yield and quality of tofu as affected by soybean and soymilk characteristics: Calcium sulfate coagulant. Journal of Food Science, 55, 1088–1092.

[fsn32770-bib-0016] Lin, T. M. , Durance, T. D. , & Scaman, C. H. (1998). Characterization of vacuum microwave, air and freeze dried carrot slices. Food Research International, 31, 111–117.

[fsn32770-bib-0017] Merrill, A. L. , & Watt, B. K. (1973). Energy value of foods: basis and derivation (Agriculture Handbook No. 74.). US Government Printing Office.

[fsn32770-bib-0018] Obatolu, V. A. (2007). Effect of different coagulants on yield and quality of tofu from soymilk. European Food Research and Technology, 226, 467–472.

[fsn32770-bib-0019] Omogbai, B. A. , & Jacob, I. B. (2013). Groundnut milk fermentation for yogurt production: Physicochemical and microbial changes. NISEB J, 13, 67–72.

[fsn32770-bib-0020] Orsat, V. , Changrue, V. , & Raghavan, G. S. V. (2006). Microwave drying of fruits and vegetables. Stewart Postharvest Review, 2, 1–7.

[fsn32770-bib-0021] Ozcan‐Sinir, G. , Ozkan‐Karabacak, A. , Tamer, C. E. , & Copur, O. U. (2019). The effect of hot air, vacuum and microwave drying on drying characteristics, rehydration capacity, color, total phenolic content and antioxidant capacity of Kumquat (*Citrus japonica*). Food Science and Technology, 39(2), 475–484. 10.1590/fst.34417

[fsn32770-bib-0022] Priya, J. A. , & Chaturvedi, A. (2020). Rapid method for detection of aflatoxin presence in groundnut by bioanalyser. In: V. Sivasubramanian , A. Pugazhendi , & I. Moorthi (eds) Sustainable development in energy and environment. Springer Proceedings in Energy. Springer, Singapore. 10.1007/978-981-15-4638-9_11

[fsn32770-bib-0023] Ranganna, S. (1999). Handbook of Analysis and quality control for Fruits and Vegetables Product, 2nd ed. Tata McGraw‐Hill Publishing Company Limited New Delhi.

[fsn32770-bib-0024] Shen, C. F. , DeMAN, L. , Buzzell, R. I. , & DeMAN, J. M. (1991). Yield and quality of tofu as affected by soyabean and soymilk characteristics: Glucono‐delta‐lactone coagulant. Journal of Food Science, 56, 109–112.

[fsn32770-bib-0025] Sumnu, G. , Turabi, E. , & Oztop, M. (2005). Drying of carrots in microwave and halogen lamp‐microwave combination ovens. LWT ‐ Food Science and Technology, 38, 549–553.

[fsn32770-bib-0026] Tan, Q. L. P. , Thanh, H. , & Thu, N. (2013). Determination of factors effecting on the hardness of soft peanut tofu using screening model. Emirates Journal of Food and Agriculture, 25, 97.

[fsn32770-bib-0027] Wang, Z. , Sun, J. , Chen, F. , Liao, X. , & Hu, X. (2007). Mathematical modeling on thin layer microwave drying of apple pomace with and without hot air pre‐drying. Journal of Food Engineering, 80, 536–544.

[fsn32770-bib-0028] Yousif, A. N. , Scaman, C. H. , Durance, T. D. , & Girard, B. (1999). Flavor volatiles and physical properties of vacuum‐microwave‐ and air‐dried sweet basil (*Ocimum* *basilicum* L.). Journal of Agricultural and Food Chemistry, 47, 4777–4781. 10552889 10.1021/jf990484m

[fsn32770-bib-0029] Zhou, J. , Yang, X. , Zhu, H. , Yuan, J. , & Huang, K. (2018). Microwave drying process of corns based on double‐porous model. Drying Technology, 37(1), 92–104. 10.1080/07373937.2018.1439952

